# Systematic Review of Antimicrobial Drug Prescribing in Hospitals

**DOI:** 10.3201/eid1202.05145

**Published:** 2006-02

**Authors:** Peter Davey, Erwin Brown, Lynda Fenelon, Roger Finch, Ian Gould, Alison Holmes, Craig Ramsay, Eric Taylor, Phil Wiffen, Mark Wilcox

**Affiliations:** * University of Dundee Medical School, Dundee, United Kingdom;; † Ninewells Hospital, Dundee, United Kingdom;; ‡ Frenchay Hospital, Bristol, United Kingdom;; § St Vincent's University Hospital, Dublin, Ireland;; ¶ Nottingham City Hospital, Nottingham, United Kingdom;; # University of Nottingham, Nottingham, United Kingdom;; ** Aberdeen Royal Infirmary, Aberdeen, United Kingdom;; †† Hammersmith Hospital, London, United Kingdom;; ‡‡ University of Aberdeen Health Services Research Unit, Aberdeen, United Kingdom;; §§ Inverclyde Royal Hospital, Greenock, United Kingdom;; ¶¶ United Kingdom Cochrane Centre, Oxford, United Kingdom;; ## Leeds General Infirmary, Leeds, United Kingdom;; *** University of Leeds, Leeds, United Kingdom

**Keywords:** Antimicrobial drug policy, antimicrobial drug resistance, Clostridium difficile, hospital-acquired infection, systematic review, synopsis

## Abstract

Prudent prescribing of antimicrobial drugs to hospital inpatients may reduce incidences of antimicrobial drug resistance and healthcare-associated infection. We reviewed the literature from January 1980 to November 2003 to identify rigorous evaluations of interventions to improve hospital prescribing of antimicrobial drugs. We identified 66 studies with interpretable data, of which 16 reported 20 microbiologic outcomes: gram-negative resistant bacteria, 10 studies; *Clostridium difficile*–associated diarrhea, 5 studies; vancomycin-resistant enterococci, 3 studies; and methicillin-resistant *Staphylococcus aureus*, 2 studies. Four studies provided strong evidence that the intervention changed microbial outcomes with low risk for alternative explanations, 8 studies provided less convincing evidence, and 4 studies provided no evidence. The strongest and most consistent evidence was for *C. difficile*–associated diahrrea, but we were able to analyze only the immediate impact of interventions because of nonstandardized durations of follow-up. The ability to compare results of studies could be substantially improved by standardizing methods and reporting.

## Abstract 2

Standardizing methods and reporting could improve interventions that reduce *Clostridium difficile*–associated diarrhea and antimicrobial drug resistance.

Despite strenuous efforts to control antimicrobial drug use and promote optimal prescribing, practitioners continue to prescribe excessively; it is estimated that up to 50% of antimicrobial drug use in hospitals is inappropriate (*1**–**3*). Antimicrobial drug resistance is largely a consequence of the selective pressures of antimicrobial drug use. Reducing these pressures by the judicious administration of these drugs should facilitate a return of susceptible bacteria or, at least, prevent or slow the pace of the emergence of drug-resistant strains (*4**,**5*). Furthermore, *Clostridium difficile*–associated diarrhea (CDAD) is a hospital-acquired infection associated with use of antimicrobial drugs (*6**–**8*) and reducing the incidences of CDAD is an additional potential benefit of improving hospital antimicrobial drug prescribing.

Implementing and monitoring interventions to optimize prescribing of antimicrobial drugs place a burden on hospital resources and their efficacies need to be confirmed (*9*). We have conducted a systematic review of interventions to improve antimicrobial drug–prescribing practices for hospital inpatients using the methods of the Cochrane Effective Practice and Organization of Care Group to assess validity (*10*). In this study, our primary objective was to evaluate the impact of interventions on reducing the incidence of colonization with or infection caused by antimicrobial drug–resistant pathogens or CDAD. In addition to the usual threats to the validity of interventions to change health care, infection control interventions are particularly prone to regression to the mean (*11*). This refers to the natural tendency of extreme observations to return towards the average (mean) over time. An epidemic or outbreak is a sequence of unusually large number of cases of infection, so that the natural history of an epidemic is to increase, peak, and then decrease. Consequently, regression to the mean is always a threat to the validity of evaluations of unplanned interventions that are initiated in response to an outbreak.

## Methods

The full protocol is available in the Cochrane Library (*10*). We searched Medline, EMBASE, the Cochrane database, and the Effective Practice and Organisation of Care specialized register for studies from January 1, 1980, to November 30, 2003, relating to prescribing of antimicrobial drugs to hospital inpatients. Additional studies were obtained from the bibliographies of retrieved articles, the Scientific Citation Index, and personal files. We requested additional data from the authors when necessary. There were no language limitations for the literature review. We included all randomized and controlled clinical trials (RCT/CCT, designs where allocation to the intervention is determined either by an explicit random process [RCT] or by a nonrandom process such as date of birth or case note number) before and after studies (a design with contemporaneous data collection before and after the intervention and an appropriate control site or activity) and interrupted time series (ITS, a clearly defined point in time when the intervention occurred and at least 3 data points before and 3 after the intervention). Data about microbiologic outcomes were considered reliable if they met the same criteria. For example, if a paper included prescribing data that met the criteria for an ITS but provided only mean data about microbiologic outcomes before and after the intervention, then the microbiologic data were not considered reliable. Two reviewers independently extracted data and assessed the quality of each study with the standardized criteria.

### Statistical Considerations

Many statistical methods can be used to analyze ITS designs (e.g., ARIMA modeling or time series regression). However, the design is often analyzed inappropriately, which makes interpretation of individual studies difficult (*12*). Methods of analyzing ITS data were examined critically (*12*). The preferred method for short time series is segmented time series regression analysis, which is a statistical comparison of time trends before and after the intervention to identify either an immediate change in the level of the regression line or a sustained change in the slope of the line (*12**,**13*). In this report, we have distinguished 2 intervention effects: immediate (a sudden change in the level of the regression line at the point of intervention) and sustained (a sustained change in the slope of the regression line from the start of the intervention phase). If the original report did not include an appropriate analysis, data were reanalyzed by using segmented time series regression.

The following model was specified: *Y*_t_ = *B*_0_ + *B*_1_ × preslope + *B*_2_ × postslope + *B*_3_ × intervention + *e*_t_, where *Y*_t_ is the outcome (e.g., CDAD incidence) in month *t*, preslope is a continuous variable indicating time from the start of the study up to the last point in the preintervention phase and coded constant thereafter, postslope is coded 0 to and including the first point postintervention and coded sequentially from 1 thereafter, and intervention is coded 0 for preintervention time points and 1 for postintervention time points. In this model, *B*_1_ estimates the slope of the preintervention data, *B*_2_ estimates the slope of the postintervention data, and *B*_3_ estimates the change in level of outcome as the difference between the estimated first point postintervention and the extrapolated first point postintervention if the preintervention line was continued into the postintervention phase. The difference in slope is calculated by *B*_2_ – *B*_1_. The error term *e*_t_ was assumed to be first-order autoregressive. Confidence intervals (95%) were calculated for all effect measures.

Formal metaanalysis of results was not attempted given the differences in context, setting, and type of outcomes. However, to gain an overall summary picture of the heterogeneity of effect sizes we standardized all measures so that they were all on the same scale. To do this, we divided the change in level and the change in slope by the preintervention standard deviation (SD) in each study. The resulting metric has no unit, it is known in standard metaanalysis as the standardized mean difference. Standardized effect sizes of 2 to 3 SD were considered large, whereas an effect size <0.5 SD was considered of questionable clinical significance even if statistically significant (*14*). To visually display the heterogeneity of the standardized effect sizes, graphic plots of level effects versus slope effects for each study (with associated 95% confidence intervals) were generated.

### Other Criteria for Assessing Evidence

The statistical analysis assessed how likely it was that study results could simply have happened by chance, and the Cochrane quality criteria assessed common threats to the validity of interventions to change practice or organization of care. To assess other threats to the validity of infection control interventions, we used the format for reporting the results of included studies recommended by guidelines derived from a recent systematic review of isolation measures to control methicillin-resistant *Staphylococcus aureus* (MRSA) (*15*). We required studies to provide reliable data about the effect of interventions on both microbial and drug outcomes with clear case definition, description of infection control measures, and other variables such as bed occupancy or staffing levels that could provide plausible alternative explanations for changes in microbial outcomes. We have provided a summary of detailed information from the included studies (Table A1). Additional information is available from the British Society for Antimicrobial Chemotherapy (http://www.bsac.org.uk). We classified case definitions into colonization, infection or clinical isolates, or a combination of >2 with the following definitions.

Colonization was defined as a microorganism, usually detected by screening, at a host site (normally nonsterile, although the urine of a catheterized patient may be an exception) without causing systemic signs of infection or a specific immune response. Colonization by case note review was established by excluding infection diagnosed according to criteria adopted by the authors or defined by appropriate bodies, e.g., the Centers for Disease Control and Prevention criteria for diagnosing nosocomial infections. Infection was established by case note review according to criteria adopted by the authors or defined by appropriate bodies or by recording specific symptoms and/or signs, such as diarrhea in patients with CDAD. Clinical isolates were defined as the recovery of a microorganism after culture of a clinical (not screening) specimen without specifying whether it represents colonization or infection.

## Results

We identified 66 intervention studies to improve prescribing of antimicrobial drugs to hospital inpatients that met our inclusion criteria (*16*) and excluded 243 studies that were uncontrolled before and after studies (n = 164) or inadequate ITS studies (n = 79). Of the 66 studies, 16 reported reliable data about 20 microbiologic outcomes: gram-negative resistant bacteria (GNRB), 10 studies; CDAD, 5 studies; vancomycin-resistant enterococci (VRE), 3 studies; and MRSA, 2 studies (Table A1). The setting for the intervention was the entire hospital in 8 studies (*17**–**24*), a single service in 2 studies (*25**,**26*), and a unit or ward in 6 studies (*27**–**32*). One intervention was educational with advice about changes in antimicrobial drugs (*17*); the other 15 interventions were restrictive (Table A1). Two studies were RCTs (*31**,**32*) and 1 study was a CCT (*30*); the remaining 13 studies used an ITS design.

### Statistical Validity

All 3 clinical trials reported appropriate statistical analysis (*30**–**32*), whereas only 2 of the 13 ITS studies reported appropriate statistical analysis (*17**,**27*). Of the remaining 11 ITS studies, 5 did not report statistical analysis; 6 reported inappropriate statistical analysis by using tests such as χ^2^ or *t* tests that assume independence between observations and do not account for time trends. Data from these 11 studies were reanalyzed.

### Effectiveness of Interventions

Overall, 4 studies provided strong evidence of control of the microbial outcome by change in prescribing (*17**,**27**,**30**,**31*). All of these studies provided reliable data about antimicrobial drug prescribing, with significant changes in both microbial and drug outcomes after planned interventions. In addition, 2 studies provided further protection against regression to the mean by using a crossover design (*27**,**30*). Three of these studies have rigorous case definitions based on prospective screening cultures plus full description of infection control measures.

Eight studies provided less convincing evidence. Two studies showed significant changes in prescribing that were associated with nonsignficant changes in CDAD (*20**,**26*). An additional 6 studies reported statistically significant improvement in microbial outcome but without reliable data about the effect of the intervention on prescribing (*18**,**19**,**23**,**24**,**28**,**29*). The importance of this omission is confirmed by the 6 studies that included reliable data about prescribing because all showed that there was some prescription of restricted drugs during the intervention phase (*17**,**20**,**26**,**27**,**30**,**31*).

Four studies had negative results (*21**,**22**,**25**,**32*). One study provided strong evidence of failure to control microbial outcomes despite a successful change in prescribing (*32*). One study reported an intervention that failed to change use of vancomycin (*22*). The remaining 2 studies showed no change in microbial outcome but did not provide reliable data about the effect of the intervention on prescribing (*21**,**25*).

### CDAD

The most consistent evidence was for the 5 interventions designed to reduce the incidence of CDAD. Four were implemented throughout the hospital (*17**,**18**,**20**,**24*) and 1 was implemented in the elderly care service (*26*); all 5 targeted prescribing of cephalosporin or clindamycin. All of the interventions were associated with a change in the expected direction (Figure part A), which was a change in the incidence of CDAD in the same direction to a change in use of cephalosporin or clindamycin. For 1 intervention, the expected direction was an increase in CDAD incidence after the introduction of ceftriaxone (*20*); for all other interventions a decrease in CDAD incidence was expected to accompany a decrease in use of cephalosporin or clindamycin. These 5 studies reported 7 interventions. The immediate effect after 6 of the 7 interventions was at least 0.5 SDs; 5 of these 7 immediate effects were statistically significant (Figure, part A). Sustained changes after the intervention were more modest, but all were in the expected directions and 4 of 7 were statistically significant (Figure, part A). The 5 CDAD studies had results expressed in different units: cases per month (*24**,**26*); cases per quarter (*18**,**20*); or cases per 1,000 admissions per year (*17*). Consequently, we were only able to compare effect sizes in numbers of CDAD cases per quarter by recalculating results from 2 studies (*24**,**26*). The antimicrobial drug intervention was associated with a mean immediate reduction of 15.0 CDAD cases per quarter (range 6–26) and a median sustained reduction of 3.2 CDAD cases per quarter (range 1–6).

**Figure:**
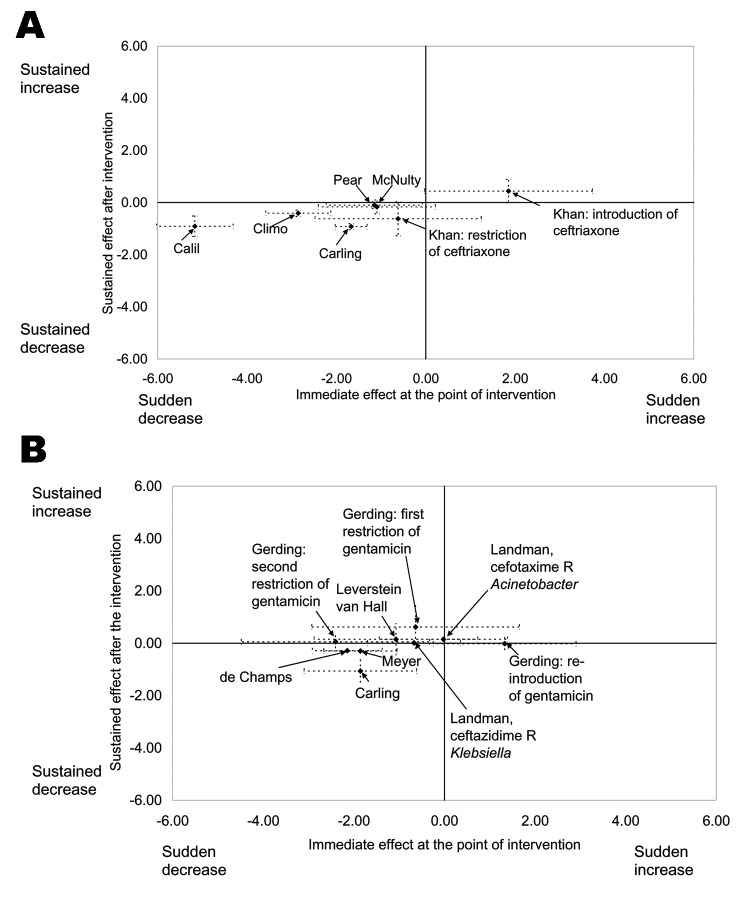
A) Standardized immediate and sustained effects for *Clostridium difficile*–associated diarrhea. B) Standardized immediate and sustained effects for resistant gram-negative bacteria.

### Resistant Gram-negative Bacteria

The results of the 10 interventions designed to reduce the incidences of GNRB were less consistent. Three were implemented throughout the hospital (*19**,**21**,**23*), 1 was implemented in the neurology and neurosurgery service (*25*), and 5 were implemented in a single intensive care unit (ICU), which included 4 with pediatric patients (*28**–**30**,**32*) and 1 with adult patients (*31*). One intervention was designed to reduce the duration of treatment with any antimicrobial drug for ICU patients at low risk for pneumonia; this was associated with a significant reduction in the incidence of colonization by any GNRB and exposure to antimicrobial drugs (*31*). The remaining 9 interventions involved changes in antimicrobial drug treatment, mainly aminoglycosides or cephalosporins. One RCT provided no evidence that antimicrobial drug cycling reduced the incidence of GNRB in a neonatal ICU (*32*). The 8 ITS studies reported 9 outcomes (Figure, part B). The expected direction of effect from a change in aminoglycoside or cephalosporin prescribing was usually a reduction in GNRB. For 1 intervention, the expected direction of effect was an increase in the incidence of GNRB after gentamicin was reintroduced (*19*). The expected direction for all 9 outcomes changed, but the effect size was <0.5 SD in 2 studies and not statistically significant in 5 studies (Figure, part B). In 3 studies the changes in slope were in the expected direction and in 1 the changes were both statistically significant and >0.5 SD, which is likely clinically important. Unlike with CDAD data, effects cannot be expressed in a common unit. Some studies measured colonization and others examined infection. Units of measurement were also variable (e.g., number of isolates, percentage of isolates, number of cases, and number of cases per time period).

### Gram-positive Bacteria

Data for gram-positive bacteria were very limited. One study provided strong evidence that restricting ceftazidime in a hematology unit was associated with significant reduction in risk for colonization by VRE (*27*). However, reduction of cephalosporin use in a hospital was not associated with any change in the prevalence of VRE isolates (*17*). A third study targeted at VRE showed that implementation of a vancomycin order form had no significant impact on vancomycin prescribing, with a trend in the unintended direction (*22*). Two studies report effects on MRSA prevalence (*17**,**21*). Our segmented regression analysis showed no significant change in response to a reduction in use of third-generation cephalosporins (Table A1), although 1 of the reports claimed that a change did occur (*21*).

## Discussion

Our primary conclusion is that 4 of the 16 studies provided strong evidence that changes in prescribing antimicrobial drugs to hospital inpatients can improve microbial outcomes (*17**,**27**,**30**,**31*). Eight of the remaining studies provided some evidence that antimicrobial drug–prescribing interventions can improve microbial outcomes, but flaws in their design indicated that there were plausible alternative explanations for the results (*18**–**20**,**23**,**24**,**26**,**28**,**29*). The remaining 4 studies were unequivocally negative (*21**,**22**,**25**,**32*).

Estimation of overall effect size was only possible for reduction in CDAD, where the evidence suggested that restriction of clindamycin or third-generation cephalosporins resulted in an immediate reduction in prevalence by 15 cases per quarter, with an additional sustained reduction by 3 cases per quarter. Prevalence is usually adjusted for clinical activity, e.g., cases per 1,000 admissions per quarter (*7*), but only 1 study provided this information (*17*). Furthermore, potentially important differences in the case definitions of CDAD occurred between the studies in our review.

Finding valid studies required painstaking analysis of a huge volume of literature, most of which is fundamentally flawed (*16*). The included studies could be dramatically improved by following guidelines for standardized reporting (*15*). In particular, the unequal duration of postintervention phases made it difficult to reliably compare the sustained effects of interventions, these being the most important outcome measures. The short and unequal duration of preintervention phases provides limited information about underlying preintervention trends. To understand how much of a change in prescribing is required to change outcome, the intervention must be independent of other control measures and be accompanied by reliable data about both prescribing and microbial outcomes.

Only 1 of the interventions was designed to reduce overall exposure to antimicrobial drugs (*31*). All of the other studies targeted the choice of antimicrobial drug (e.g., by restricting access to third-generation cephalosporins in favor of drugs recommended by the hospital antimicrobial drug policy) but did not aim to shorten the duration of treatment. This intervention (*31*) shortened the duration of antimicrobial drug treatment for ICU patients at low risk for ventilator-associated pneumonia. This study was conducted in an ICU with adult patients. However, the same principle of using clinical scores to identify low-risk patients, in whom antimicrobial drug therapy could be stopped, has been developed in other clinical settings (*33**–**35*), and the impact on microbiologic outcomes should be investigated.

None of the studies provided evidence for cost-effectiveness or clinical outcome. The study designs likely did not have sufficient power to measure these outcomes. Few studies provided data about multiple microbiologic species and 1 of these endpoints (incidence of cefotaxime-resistant *Acinetobacter* spp.) was opposite to that which was expected (*21*). Future studies should provide more data about cost and clinical outcomes. Notably, evidence is needed to show that interventions do not have adverse outcomes.

The potential for the success of antimicrobial drug interventions likely varies by organism (*36**,**37*). Antimicrobial drugs are likely to play a large role in the selection of enterobacteria expressing extended-spectrum β-lactamases, a minimal role in the selection and transmission of MRSA, and an intermediate role in VRE. However, the available evidence is not sufficient to investigate these hypotheses.

### Implications for Practice

The evidence supports the theory that limiting the use of specific antimicrobial drugs will reduce the prevalences of resistant gram-negative bacteria and CDAD. For gram-positive bacteria, there is a lack of evidence rather than evidence of no effect. Hospitals would like to know how much they should limit their antimicrobial drug prescriptions and what is the minimum that will show a real effect. Unfortunately, the available evidence is too limited to provide definitive answers to these issues. Thus, hospitals must estimate the effect of their own interventions. The good news is that the data required for ITS analysis of the incidences of drug-resistant bacteria or CDAD should be readily available in most hospitals. Healthcare providers need to invest in data analysis so that evaluation of antimicrobial drug control in hospitals becomes a routine measure of the quality of care rather than a research project.

Standardized reporting of outbreaks and interventions to control the incidence of antimicrobial drug resistance or hospital-acquired infection would greatly enhance the ability to combine results from hospitals in metaanalyses. Key issues include full description of other infection control measures, consistent and reproducible case definitions, the length of preintervention and postintervention phases, and the intervals between data points (*15*). Ideally, data should be made available in a way that allows reanalysis and, where appropriate, metaanalysis. Metaanalysis of single hospital studies is no substitute for good multicenter studies, but it could be used to provide some evidence of reproducibility and thus to prioritize targets for definitive trials.

### Priorities for Research

The research agenda needs to move to multicenter studies with randomized allocation to interventions. This will provide better evidence of external validity as well as the power to measure cost-effectiveness and exclude important unintended adverse clinical outcomes. Development and pilot testing of the effectiveness of clinical decisions for reducing unnecessary exposure to antimicrobial drugs should be a priority for research in hospitals.

## Appendix

**Table A1:** Summary of included studies* (Download PDF (74 KB, 5 pages)

Study, y (reference)	Setting and population	Design	Main interventions	Outcomes	Assessment of evidence
Bradley et al., 1999 (27)	Adult hematology unit in UK, 261 patients who were not carriers of VRE at the start of the study	Prospective ITS with 3 phases of 4, 6, and 5 months. Planned intervention. Case definition: colonization. Other infection control measures consistent through study.	Phase 1: ceftazidime for empiric antimicrobial drug treatment Phase 2: antimicrobial drug policy changed to piperacillin tazobactam. Phase 3: antimicrobial drug policy changed back to ceftazidime.	Microbial: % of patients colonized with VRE fell from 57% in Phase 1 to 19% in Phase 2, then rose again to 36% in Phase 3: significant by log rank test. Drug: significant reduction in ceftazidime use in Phase 2: immediate –227.8 patient days per month, p<0.001; sustained –19.3 patient days per month, p = 0.037.	Statistically significant reduction in risk of colonization with VRE associated with reduction in antimicrobial drug prescribing. No major weaknesses in the study design.
Calil et al., 2001 (28)	Neonatal unit in Brazil, 342 patients in a 30-bed unit (8 intensive care and 22 intermediate care beds)	Prospective ITS with 2 phases of 3 months each. Unplanned intervention. Case definition: colonization. Other infection control measures were introduced during the study and it is not clear how they related to the antimicrobial drug intervention.	Phase 1: usual care. Phase 2: implementation of infection control measures emphasizing hand washing and contact precautions plus an antimicrobial drug policy restricting use of third-generation cephalosporins.	Microbial: cases of multi-resistant *Enterobacter cloacae* colonization per month decreased in Phase 2: immediate –15.51 cases per month (p = 0.054); sustained –2.73 cases per month (p = 0.138). Drug: no reliable data.	Significant reduction in colonization but it is not possible to separate the effects of the infection control measures from the change in antimicrobial drug policy. Several other potentially important weaknesses.
Carling et al., 2003 (17)	Single medium-sized community teaching hospital (affiliated with a University) in US. No obstetric unit or pediatric ICU.	Hybrid retrospective and prospective ITS with 2 phases of 36 and 84 months. Planned intervention. Case definition: infection with CDAD or resistant gram-negative bacteria, MRSA, or VRE. Other infection control measures consistent through study.	Phase 1: automatic 7-day stop order on all antimicrobial drugs, limited reporting of susceptibility tests, and educational program. Phase 2: as Phase 1 plus review of patients receiving target antimicrobial drugs by pharmacist and ID physician, recommendations placed in the case notes.	Microbial: CDAD and resistant *Enterobacteriaceae* in cases per 1,000 admissions. MRSA and VRE as % clinical isolates. Postintervention: there were significant reductions for CDAD: immediate –1.47 cases, p<0.001; sustained –0.81 cases, p = 0.05. Resistant Enterobacteriaceae also reduced: immediate –2.34 cases, p = 0.03; sustained –1.34 cases, p = 0.01. There was no significant change in the % isolates of MRSA or VRE. Drug: authors' regression analysis shows significant reduction in target antimicrobial drugs in Phase 2.	Significant reduction in CDAD cases and resistant Enterobaceriaceae associated with planned antimicrobial drug intervention that resulted in significant changes in antimicrobial drug use. Main weaknesses were the lack of detail about infection control and the case definition for resistant *Enterobacteriaceae.*
Climo et al., 1998 (18)	Single 703-bed tertiary care hospital in USA	Hybrid retrospective and prospective ITS with 2 phases of 27 and 33 months. Unplanned intervention. Case definition: infection, CDAD. Other infection control measures consistent through study.	Phase 1: infection control only Phase 2: infection control plus restriction of clindamycin.	Microbial: CDAD cases per quarter. The intervention was associated with significant reduction in CDAD cases per quarter: immediate –26.3 cases, p<0.001; sustained –3.8 cases, p<0.001. Drug: no reliable data.	Significant reduction in CDAD cases in phase 2. However, this was an unplanned intervention, there were no reliable data about drug use, and the study had several other potentially important weaknesses.
de Champs et al., 1994 (29)	Single pediatric ICU with 15 ventilator beds and 28 intermediate-care beds in France	Prospective ITS with 2 phases of 7 and 12 months. Unplanned intervention. Case definition: infection by resistant *E cloacae*. Other infection control measures consistent through study.	Phase 1: barrier precautions only. Phase 2: barrier precautions plus removal of gentamicin from the unit and replacement with amikacin.	Microbial: The intervention was associated with significant reduction in resistant *E cloacae* cases per month, immediate –7.47 cases, p<0.001; sustained –1.00 cases, p = 0.002. Drug: no reliable data.	Significant reduction in *E cloacae* cases in phase 2. However, this was an unplanned intervention, there were no reliable data about drug use, and the study had several other potentially important weaknesses.
de Man et al., 2001 (30)	Two similar neonatal ICUs in the same hospital. The study enrolled 436 patients with a mean of 33 weeks gestation.	Prospective cluster controlled clinical trial with crossover with 2 phases of 6 months each. Planned intervention. Case definition: colonization plus clinical isolates. Other infection control measures: consistent through study.	Phase 1: unit A used amoxicillin plus cefotaxime, unit B used penicillin plus tobramycin. Phase 2: antimicrobial drug policies were switched: unit A used penicillin plus tobramycin, unit B used amoxicillin plus cefotaxime.	Microbial: the cefotaxime and amoxicillin regimen was associated with a relative risk of colonization by gram-negative bacteria resistant to cefotaxime or tobramycin of 2.98 (95% CI 1.64–5.38). Drug: cefotaxime plus amoxicillin exposure was 26%–32% of patient days when that regimen was in place *vs.* 1% when penicillin plus tobramycin was used.	Significantly increased risk of colonization associated with the cefotaxime and amoxicillin regimen. However, risk of colonization was also related to length of stay and was significantly shorter in the penicillin plus tobramycin phase.
Gerding et al., 1985 (19)	Single Veterans Administration hospital in US	Prospective ITS with 4 phases of 4, 26, 12, and 12 months. Planned intervention. Case definition: clinical isolates. Other infection control measures not described.	Phase 1: no restriction. Phase 2: gentamicin restricted. Phase 3: amikacin restricted. Phase 4: gentamicin restricted.	Microbial: % of all gram-negative aerobic bacilli resistant to gentamicin. Figure shows resistance to gentamicin varied between 15% and 2% over the study, falling and rising with no clear relationship to changes in antimicrobial drug policy. Drug: no reliable data.	Little evidence that the fluctuations in resistance to gentamicin were related to antimicrobial drug policy changes. Several potentially important design weaknesses.
Khan and Cheesbrough, 2003 (20)	Single 800-bed nonteaching hospital in UK	Prospective ITS with 3 phases of 6, 13, and 5 months. Phase 2 planned, Phase 3 unplanned. Case definition: CDAD infection. Other infection control measures consistent through study.	Phase 1: cefotaxime. Phase 2: ceftriaxone. Phase 3: levofloxacin.	Microbial: Phase 2 was associated with increase in CDAD cases per quarter: immediate +19.7 cases, p = 0.07; sustained +4.7 cases p = 0.07. Phase 3 was associated with sustained reduction in CDAD by –5.8 cases per quarter, p = 0.08. Drug: no reliable data for Phase 1, significant reduction in ceftriaxone use (g per quarter) in Phase 3.	Non significant changes in CDAD were associated with the introduction and restriction of ceftriaxone. Regression to the mean was a plausible alternative explanation for changes in phase 3 and reliable drug data were provided only for phases 2 and 3.
Landman et al., 1990 (21)	Single university hospital in US with 569 discharges per month from medical and surgical services	Retrospective ITS with 2 phases of 29 and 23 months. Planned intervention. Case definition: clinical isolates of resistant bacteria. Other infection control measures: none specific to the bacteria under study.	Phase 1: unrestricted. Phase 2: restriction of third-generation cephalosporins, clindamycin, and vancomycin by requiring approval by an ID physician.	Microbial: intervention was not associated with a significant reduction in the incidence of either ceftazidime-resistant *Klebsiella pneumoniae* or MRSA. However, there was a significant sustained increase in cefotaxime-resistant *Acinetobacter* spp: by +0.337 new cases per 1,000 discharges. Drug: no reliable data.	The intervention was associated with a significant but unintended increase in one of the outcomes and no significant changes in the other. However, there were important weaknesses in the study design.
Lautenbach et al., 2003 (22)	Single 725-bed University hospital in US	Hybrid retrospective and prospective ITS with 2 phases of 36 and 84 months. Unplanned intervention. Case definition: clinical isolates of VRE. Other infection control measures not described.	Phase 1: unrestricted use of antimicrobial drugs. Phase 2: use of vancomycin or third-generation cephalosporins for >72 h required approval by the antimicrobial drug management team. After 24 months any use of vancomycin required approval.	Microbial: regression analysis suggests that the intervention was associated with significant reduction in % VRE but this result was an artifact caused by the first point in the data (1% VRE) and only having 3 preintervention points. Drug: no significant change in vancomycin use (DDD/1,000 patient days)	No evidence supporting control by antimicrobial drug restriction because the restriction did not reduce the use of vancomycin. No data about infection control measures and there were other important weaknesses in the study design.
Leverstein-van Hall et al., 2001 (25)	Neurology and neurosurgery wards in a single 858-bed university hospital in the Netherlands.	Prospective ITS with 2 phases of 1 and 2 months. Unplanned intervention. Case definition: colonization. Other infection control measures consistent through study but only implemented 4 weeks before the start of antimicrobial drug restriction.	Phase 1: stringent barrier precautions. Phase 2: restriction of all antimicrobial drugs by requiring approval by microbiology or ID. Only amikacin or carbapenems used for treatment of gram-negative infection.	Microbial: % prevalence of intestinal colonization by gentamicin-resistant *Enterobacteraiaceae* was decreasing preintervention: by –1.3 % per week and there was no significant change postintervention. Drug: no reliable data.	No evidence supporting control by antimicrobial drug restriction. There were several important weaknesses in the study design.
McNulty et al., 1997 (26)	Care of the elderly unit in a single nonteaching hospital in UK.	Prospective ITS with 2 phases of 7 and 16 months. Unplanned intervention. Case definition: infection, CDAD. Other infection control measures consistent through study.	Phase 1: increased ward cleaning and patient isolation. Phase 2: restriction of cephalosporins by removal from ward stock; infection control measures as in Phase 1.	Microbial: phase 2 was associated with nonsignificant reduction in CDAD: immediate –3.22, cases per month, p = 0.120; sustained –0.50 cases per month, p = 0.230. Drug: intervention was associated with significant reduction in cefuroxime cost: immediate –£501.78 per month, p = 0.015.	Nonsignificant reduction in CDAD cases. This was an unplanned intervention and the study had several other potentially important weaknesses.
Meyer et al., 1993 (23)	A single 487-bed university hospital in US	Hybrid retrospective and prospective ITS with 2 phases of 14 and 11 months. Unplanned intervention. Case definition: infection plus colonization. Other infection control measures: barrier precautions implemented at the same time as ceftazidime restriction.	Phase 1: usual care. Phase 2: barrier precautions for infected or colonized patients plus restriction of ceftazidime. Case notes were reviewed for 133 of the 142 patients with resistant isolates, of whom 52 (39%) met CDC criteria for nosocomial infection.	Microbial: number of cases of ceftazidime-resistant *K. pneumoniae* per 1,000 average daily census. Phase 2 was associated with significant reduction: immediate –38.6 cases, p<0.0001; sustained –6.2 cases, p<0.0001. Drug: drug data are provided for different periods (22 months preintervention and 6 months postintervention) but do show a significant reduction in the number of patients receiving ceftazidime: immediate –26.4 patients, p =0.003; sustained ­–10.21 patients, p<0.001.	Significant reduction in ceftazidime-resistant *K. pneumoniae* in phase 2 with significant reduction in ceftazidime use. However, it is impossible to separate the effect of ceftazidime restriction from the infection control measures. Regression to the mean was another plausible explanation.
Pear et al., 1994 (24)	A single university hospital in the US with an average daily census of 168 patients	Hybrid retrospective and prospective ITS with 2 phases of 40 and 14 months. Unplanned intervention. Case definition: infection, CDAD. Other infection control measures consistent across study.	Phase 1: hospital staff education, increased use of gloves and improved environmental hygiene. Phase 2: restriction of clindamycin by prior approval by ID physician; infection control measures maintained as in Phase 1.	Microbial: number of CDAD cases per month. Phase 2 was associated with significant reduction, immediate –3.68 cases per month, p = 0.041, sustained –0.32 cases per month, p = 0.134). Drug: no reliable data.	Significant reduction in CDAD in phase 2 but this was an unplanned intervention and there were no reliable data about drug use.
Singh et al., 2000 (31)	Adult surgical and medical ICUs in a single university-affiliated Veterans Administration hospital in US. 81 patients included, mean age 69 years.	Randomized trial with followup of patients until they were discharged from ICU or died. Planned intervention. Case definition: colonization plus clinical isolates. Other infection control measures not described but it is reasonable to assume that they were consistent for the intervention and control patients.	Control group: choice, number, and duration of antimicrobial drugs at the discretion of the care providers. Intervention group: patients received standardized initial therapy (ciprofloxacin IV for 3 days) with assessment at 3 days when antimicrobial drugs were stopped if the patient was judged to be at low risk of pneumonia based on the CPIS score.	Microbial: % patients colonized or infected with resistant bacteria. RR for intervention *vs.* control: 0.36, 95% CI 0.14–0.89. Drug: RR of receiving antimicrobial drugs for > 3 days, intervention *vs.* control: 0.29, 95% CI 0.17–0.48. Clinical: length of ICU stay (9.4 days intervention vs. 14.7 days control; p = 0.04); Nonsignificant reduction in deaths: RR of 30-day death: 0.41, 95% CI 0.16–1.05	Statistically significant reduction in risk of colonization and infection with resistant bacteria associated with reduction in antimicrobial drug prescribing. Clinical noninferiority of the intervention regimen was confirmed. No major weaknesses.
Toltzis et al., 2002 (32)	Single 38-bed neonatal intensive care unit in a University hospital in US. 1,062 episodes of care in infants with mean age 35 weeks	Randomized trial with followup of patients until they were discharged from ICU or died. Planned intervention. Case definition: colonization. Other infection control measures not described but it is reasonable to assume that they were consistent for the intervention and control patients.	Control group: prescribing according to individual preference of physicians. Intervention group: monthly rotation between gentamicin, followed by piperacillin-tazobactam, followed by ceftazidime, followed by gentamicin again.	Microbial: % of patients colonized with resistant bacteria. RR was greater in the Intervention group: 1.40, 95% CI 0.95–2.05. Drug: control patients received predominantly gentamicin. The intervention group received the intended antimicrobial drugs on 84% of all antimicrobial days. No difference in total antimicrobial drug use. Clinical: all cause death was similar: 3.2% intervention vs. 2.3% control.	No evidence supporting control of resistance by antimicrobial drug cycling. No major weaknesses. The authors provide 4 alternative explanations other than failure of cycling: NICU population, rotation too rapid, inclusion of ceftazidime, use of ampicillin in all regimens.

*VRE, vancomycin-resistant enterococci; ITS, interrupted time series; ICU, intensive care unit; CDAD, *Clostridium difficile*–associated diarrhea; MRSA, methicillin-resistant *Staphylococcus aureus*; ID, infectious disease; CI, confidence interval; DDD, defined daily dose; CDC, Centers for Disease Control and Prevention; IV, intravenous; RR, relative risk; CPIS, clinical pulmonary infection score; NICU, neonatal intensive care unit. Additional information is available from http://www.bsac.org.uk
